# Supplementary material for: Projecting Lifetime Health Outcomes and Costs Associated with the Ambient Fine Particulate Matter Exposure among Adult Women in Korea
Source: Int J Environ Res Public Health. 2022 Feb 22;19(5):2494. doi: 10.3390/ijerph19052494 (PMC8909340; doi:10.3390/ijerph19052494)
Supplement: Supplementary file 1 [file ijerph-19-02494-s001.zip › ijerph-1534949-supplementary.pdf]

Study selection process

1) Lung Cancer

Step	Search history	Results
#1	woman or female	9,592,249
#2	particulate matter or pm2.5	80,735
#3	lung cancer or lung carcinoma	380,609
#4	#1 and #2 and #3	1,406
#5	#1 and #2 and #3 and (relative risk or hazard ratio) and (incidence rate or prevalence or mortality)	683
#6	Filters: English, Korean, Adult: 19+ years	430

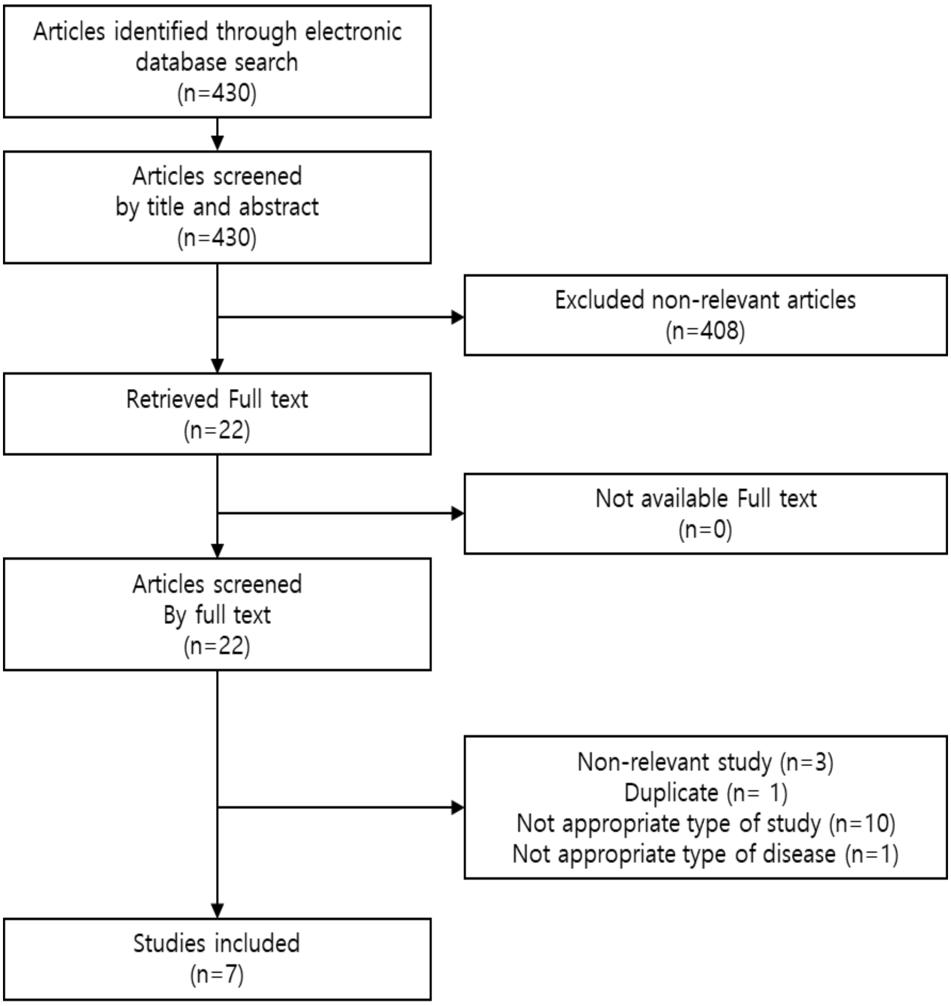

	Reference	$PM_{2.5}$ exposure (μg/m³)	Follow-up period (years)	n	Sex (Female %)	Age	Country	Outcome	$PM_{2.5}$ increment (μg/m3)	Results	
**Incidence**	Gharibvand et al(2017) [1]	12.88	7.5	80,285	58.8-65.2	57.02-68.75	US/ Canada	HR	10	1.42(1.02-1.98)	Meta analysis
	Li et al(2020) [2]	53.9-57.2	15	118,551	58.9	51	China	HR	x	1.44(1.10-1.88)	Cohort study
**Mortality**	Turner et al(2011) [3]	14	9	177,752	71.4	50-59	South America	HR	10	1.27(1.03-1.56)	Cohort study
	Cui et al(2015) [4]	10	14	4,992,425	x	25-95	US	RR	10	1.09(1.06-1.11)	SR / Meta analysis
	Huang et al(2017) [5]	x	4-34	54,663,961	x		Multinational	RR	10	1.11(1.05-1.18)	SR / Meta analysis
	Li et al(2020) [2]	53.9-57.2	15	118,551	58.9	51	China	HR	x	1.83(1.33-2.50)	Cohort study
	Pope et al(2020) [6]	10.7	20	635,539	55.5	45.3	US	HR	10	1.13(1.00-1.26)	Cohort study
	Wang et al(2020) [7]	10.3	8	988,643	x	65-120	US	HR	10	1.078(1.056-1.100)	Cohort study (retrospective)

Gharivband et al(2017) was selected because the exposure concentration and $PM_{2.5}$ increment were appropriate. Turner et al(2011) was selected because the exposure concentration was appropriate and the majority of the study population was women.

2) Myocardial Infarction

Step	Search history	Results
#1	woman or female	9,591,872
#2	particulate matter or pm2.5	80,728
#3	myocardial infarction or cardiovascular disease or ischemic heart disease or coronary heart disease	2,741,085
#4	#1 and #2 and #3	2,358
#5	#1 and #2 and #3 and (relative risk or hazard ratio) and (incidence rate or prevalence or mortality)	1,242
#6	Filters: English, Korean, Adult: 19+ years	972

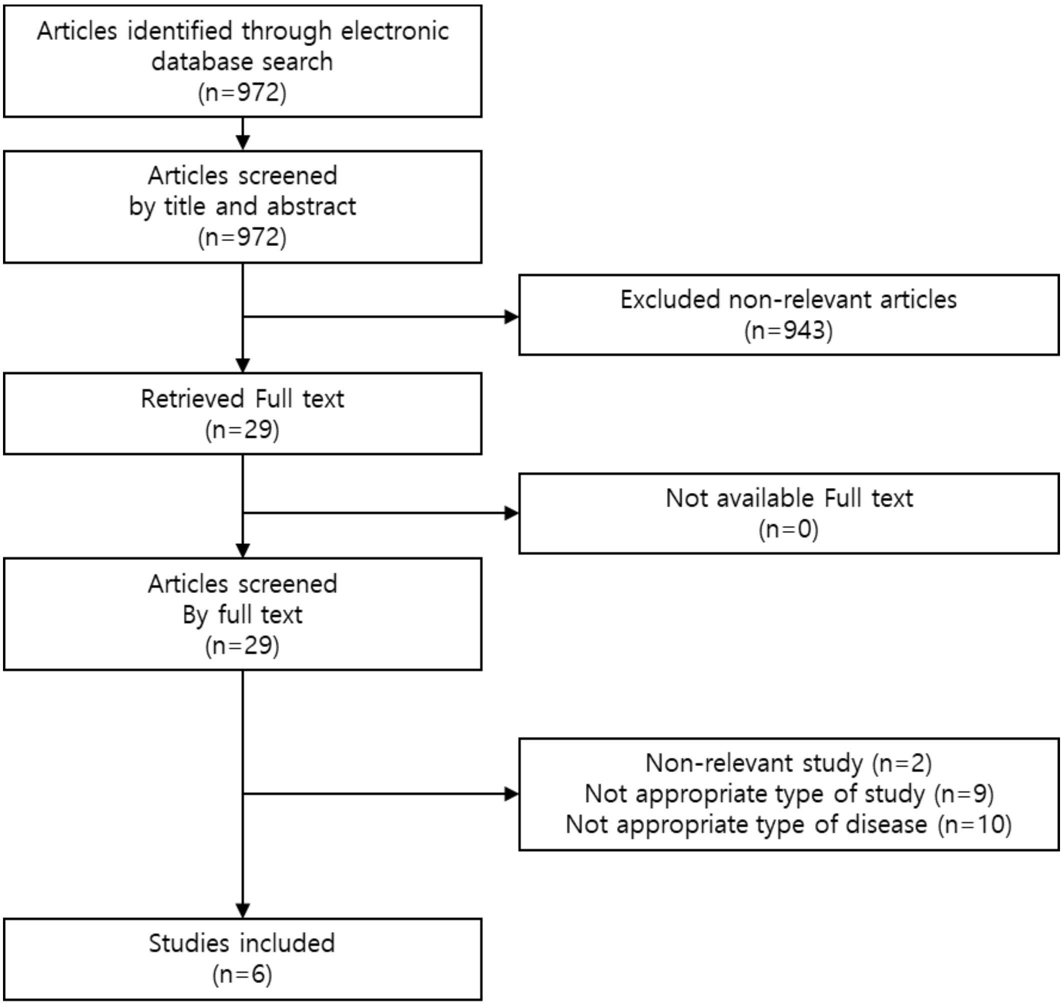

	Reference	$PM_{2.5}$ exposure ($\mu g/m^3$)	Follow-up period (years)	n	Sex (Female%)	Age	Country	Outcome	$PM_{2.5}$ increment (μg/m3)	Results	
Incidence	Miller et al(2007) [8]	13.5	6	65,893	100	62.9-63.9	US	HR	10	1.06(0.85-1.34)	Cohort study
	Lipsett et al(2011) [9]	15.64	5.6	124,614	100	Over 20	US	HR	10	0.98(0.83-1.16)	Cohort study
	Cesaroni et al(2014) [10]	7-31	11.5	100,166	48-55	44-74	Europe	HR	5	1.22(1.04-1.44)	SR / Meta-analysis
	Cramer et al.(2020) [11]	19.6	18.6	22,241	100	52.6	Denmark	HR	5.3	1.20(1.07-1.35)	Cohort study
Mortality	Puett et al(2009) [12]	7.7	10	66,250	100	62.4	US	HR	10	2.13(1.07-4.26)	Cohort study
	Lipsett et al(2011) [9]	15.64	8.3	124,614	100	Over 20	US	HR	10	1.20(1.02, 1.41)	Cohort study
	Beelen et al(2014) [13]	6.6-31	13.9	367,383	63	50.5	Europe	HR	5	1.07(0.82-1.41)	SR / Meta-analysis

Cramer et a l(2020) was selected because the exposure concentration and it had long follow-up period with large sample size. Lipsett et al(2011) was selected because of the exposure concentration, gender composition and large sample size, despite inappropriate age distribution since other studies do not fit with the $PM_{2.5}$ exposure criteria. .

3) Stroke

Step	Search history	Results
#1	woman or female	9,593747
#2	particulate matter or pm2.5	80,746
#3	myocardial infarction or cardiovascular disease or ischemic heart disease or coronary heart disease	606,977
#4	#1 and #2 and #3	446
#5	#1 and #2 and #3 and (relative risk or hazard ratio) and (incidence rate or prevalence or mortality)	291
#6	Filters: English, Korean, Adult: 19+ years	243

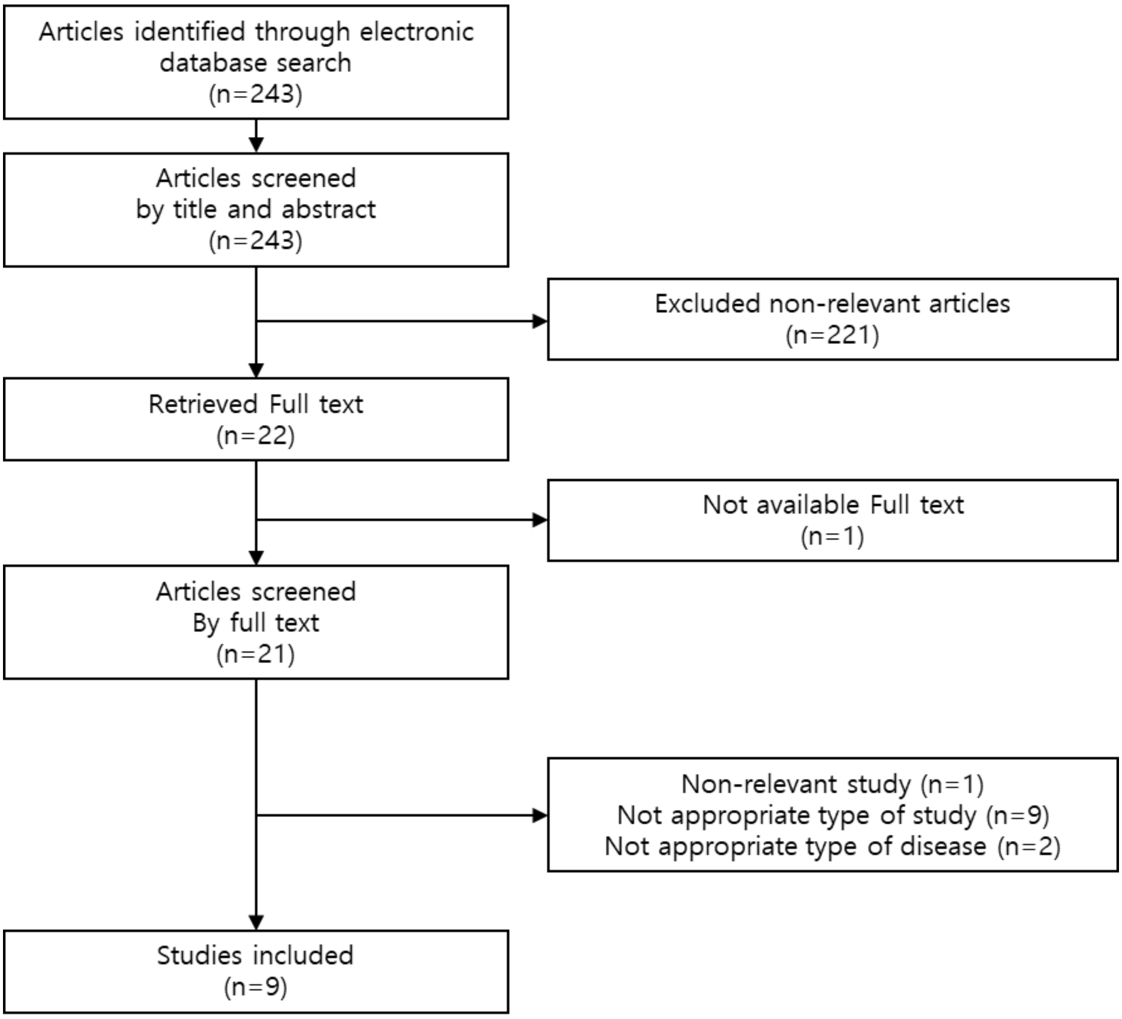

	Reference	$PM_{2.5}$ exposure (μg/m^3)	Follow-up period (years)	n	Sex (Female%)	Age	Country	Outcome	$PM_{2.5}$ increment (μg/m3)	Results	
Incidence	Miller et al(2007) [8]	13.5	6	65,893	100	62.9-63.9	US	HR	10	1.28(1.02-1.61)	Cohort study
	Lipsett et al(2011) [9]	15.64	8.3	124,614	100	Over 20	US	HR	10	1.14(0.99-1.32)	Cohort study
	To T et al (2015) [14]	x	30	29,549	100	50-54	Canada	IR	10	1.26(1.11-1.43)	Cohort study
	Huang et al(2019) [15]	64.9	23	117,575	59	50.9	China	HR	10	1.26(1.23-1.29)	Cohort study
	Noh et al(2019) [16]	25.1	12	62,676	50.7	50.7	Korea	HR	10	1.20(1.05-1.38)	Cohort study (retrospective)
	Hystad et al(2020) [17]	47.5	15	157,436	58	50.2	Multinational	HR	10	1.13(1.10-1.15)	Cohort study
Mortality	Lipsett et al(2011) [9]	15.64	8.3	124,614	100	Over 20	US	HR	10	1.16(0.92-1.46)	Cohort study
	Beelen et al(2014) [13]	6.6-31	13.9	367,383	63	50.5	Europe	HR	5	1.34(0.94-1.91)	SR / Meta analysis
	Hayes et al(2019) [18]	13.3	x	565,477	40	62.8	US	HR	10	1.14(1.02-1.17)	Cohort study
	Yang et al(2021) [19]	66.3	9.8	38,140	50.2	44	China	HR	10	1.27(1.01-1.60)	Cohort study

Miller et al(2007) was selected because the exposure concentration was appropriate and the study population was consisted of women only. Beelen et al(2014) was selected because the exposure concentration was appropriate, included large study populations with long follow-up period. Lipsett et al (2011) was not considered because of the age distribution and there was possible misclassification of disease since the study excluded women who had history of MI or stroke.

4) Chronic Obstructive Pulmonary Disease

Step	Search history	Results
#1	woman or female	9,589,797
#2	particulate matter or pm2.5	80,712
#3	chronic obstructive pulmonary disease or COPD	94,319
#4	#1 and #2 and #3	1,053
#5	#1 and #2 and #3 and (relative risk or hazard ratio) and (incidence rate or prevalence or mortality)	358
#6	Filters: English, Korean, Adult: 19+ years	269

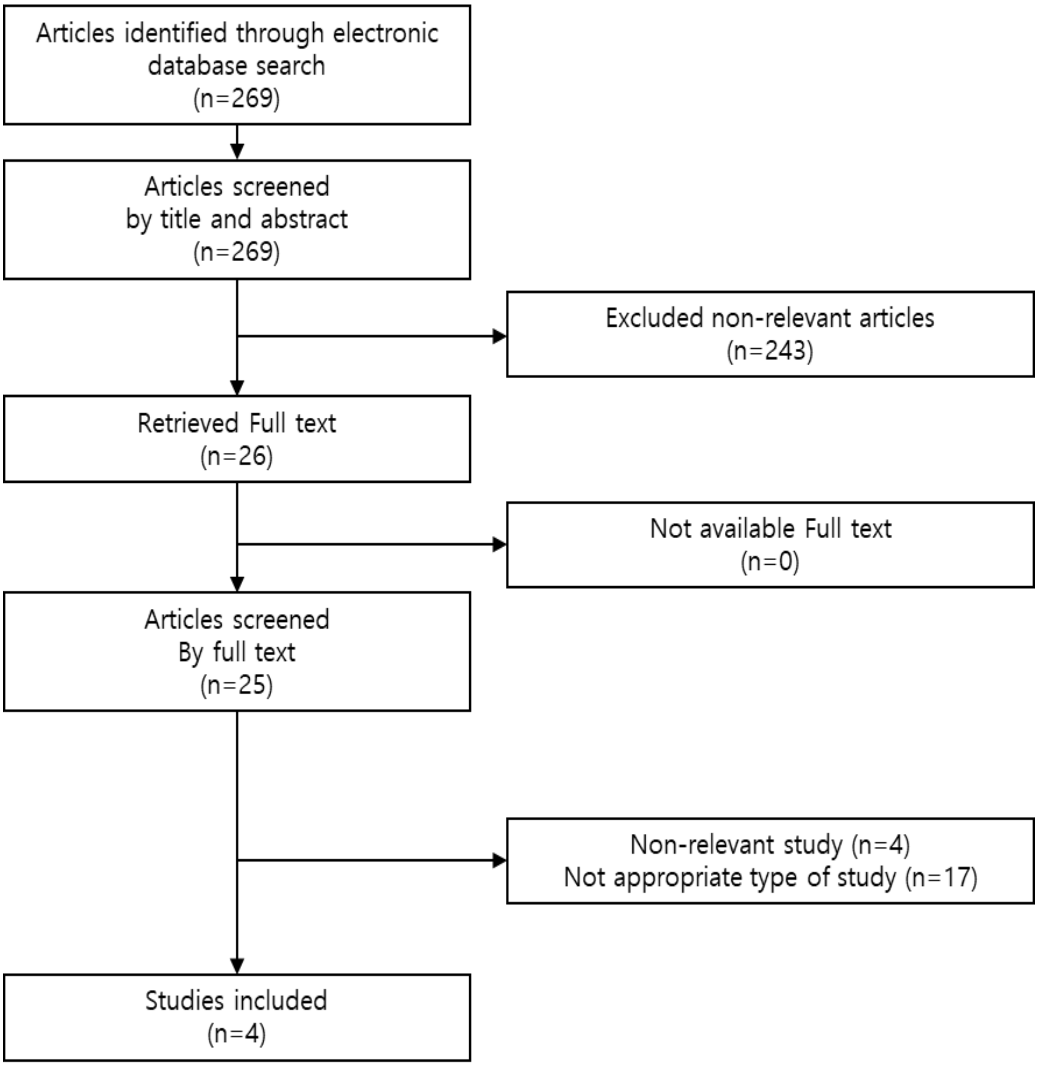

	Reference	$PM_{2.5}$ exposure (μg/m^3)	Follow-up period (years)	n	Sex (Female %)	Age	Country	Outcome	$PM_{2.5}$ increment (μg/m3)	Results	
**Incidence**	Schikowski et al(2014) [20]	8.17-34.27	25	3,643	61.7	35.1 - 54.3	Europe	OR	5	1.06(0.73-1.53)	SR / Meta analysis
	Guo et al (2018) [21]	26.91	5.9	91,709	48.8	40.3	Taiwan	HR	5	1.08(1.04-1.11)	Cohort study
**Mortality**	Pinault et al(2017) [22]	7.37	10.6	11,900	51.6	48.4	Canada	HR	10	1.238(1.106-1.386)	Cohort study
	Pun et al(2016) [23]	12.5	8	238,214	x	x	US	RR	10	1.169(1.136-1.203)	Cohort Study (retrospective)

Guo et al(2018) was selected because the sample size; yet the values from two studies are very close. Schikowski et al(2014) was not selected because the study used a back extrapolated value for the exposure data and it had uncertainties in the finding. Pun et al(2016) was selected because the exposure concentration was appropriate even though other information was insufficient. Authors conducted sensitivity analysis with different relative risks to examine the impact of each variables (Table 5, Figure 3)

REFERENCES

1. Gharibvand, L., et al., *The Association between Ambient Fine Particulate Air Pollution and Lung Cancer Incidence: Results from the AHSMOG-2 Study.* Environ Health Perspect, 2017. **125**(3): p. 378-384.
2. Li, J., et al., *Chronic Effects of High Fine Particulate Matter Exposure on Lung Cancer in China.* American Journal of Respiratory and Critical Care Medicine, 2020. **202**: p. 1551-1559.
3. Turner, M.C., et al., *Long-term ambient fine particulate matter air pollution and lung cancer in a large cohort of never-smokers.* Am J Respir Crit Care Med, 2011. **184**(12): p. 1374-81.
4. Cui, P., et al., *Ambient particulate matter and lung cancer incidence and mortality: a meta-analysis of prospective studies.* Eur J Public Health, 2015. **25**(2): p. 324-9.
5. Huang, F., et al., *Relationship between exposure to PM2.5 and lung cancer incidence and mortality: A meta-analysis.* Oncotarget, 2017. **8**(26): p. 43322.
6. Pope, C.A., 3rd, et al., *Mortality Risk and Fine Particulate Air Pollution in a Large, Representative Cohort of U.S. Adults.* Environ Health Perspect, 2019. **127**(7): p. 77007.
7. Wang, B., et al., *The impact of long-term PM2.5 exposure on specific causes of death: exposure-response curves and effect modification among 53 million U.S. Medicare beneficiaries.* Environ Health, 2020. **19**(1): p. 20.
8. Miller, K.A., et al., *Long-Term Exposure to Air Pollution and Incidence of Cardiovascular Events in Women.* New England Journal of Medicine, 2007. **356**(5): p. 447-58.
9. Lipsett, M.J., et al., *Long-term exposure to air pollution and cardiorespiratory disease in the California teachers study cohort.* Am J Respir Crit Care Med, 2011. **184**(7): p. 828-35.
10. Cesaroni, G., et al., *Long term exposure to ambient air pollution and incidence of acute coronary events: prospective cohort study and meta-analysis in 11 European cohorts from the ESCAPE Project.* BMJ, 2014. **348**: p. f7412.
11. Cramer, J., et al., *Long-Term Exposure to Air Pollution and Incidence of Myocardial Infarction: A Danish Nurse Cohort Study.* Environ Health Perspect, 2020. **128**(5): p. 57003.
12. Puett, R.C., et al., *Chronic fine and coarse particulate exposure, mortality, and coronary heart disease in the Nurses' Health Study.* Environ Health Perspect, 2009. **117**(11): p. 1697-701.
13. Beelen, R., et al., *Long-term Exposure to Air Pollution and Cardiovascular Mortality An Analysis of 22 European Cohorts.* Epidemiology, 2014. **25**(3): p. 368-378.
14. To, T., et al., *Chronic disease prevalence in women and air pollution--A 30-year longitudinal cohort study.* Environ Int, 2015. **80**: p. 26-32.
15. Huang, K., et al., *Long term exposure to ambient fine particulate matter and incidence of stroke: prospective cohort study from the China-PAR project.* BMJ, 2019. **367**: p. l6720.
16. Noh, J., et al., *Long-term Effects of Cumulative Average PM2.5 Exposure on the Risk of Hemorrhagic Stroke.* Epidemiology, 2019. **30 Suppl 1**: p. S90-S98.
17. Hystad, P., et al., *Associations of outdoor fine particulate air pollution and cardiovascular disease in 157 436 individuals from 21 high-income, middle-income, and low-income countries (PURE): a prospective cohort study.* The Lancet Planetary Health, 2020. **4**(6): p. e235-e245.
18. Hayes, R.B., et al., *PM2.5 air pollution and cause-specific cardiovascular disease mortality.* Int J Epidemiol, 2020. **49**(1): p. 25-35.
19. Yang, X., et al., *Long-term exposure to ambient PM2.5 and stroke mortality among urban residents in northern China.* Ecotoxicol Environ Saf, 2021. **213**: p. 112063.
20. Schikowski, T., et al., *Association of ambient air pollution with the prevalence and incidence of COPD.* Eur Respir J, 2014. **44**(3): p. 614-26.
21. Guo, C., et al., *Effect of long-term exposure to fine particulate matter on lung function decline and risk of chronic obstructive pulmonary disease in Taiwan: a longitudinal, cohort study.* The Lancet Planetary Health, 2018. **2**(3): p. e114-e125.
22. Pinault, L.L., et al., *Associations between fine particulate matter and mortality in the 2001 Canadian*

Census Health and Environment Cohort. Environ Res, 2017. **159**: p. 406-415.
23. Pun, V.C., et al., *Long-Term PM2.5 Exposure and Respiratory, Cancer, and Cardiovascular Mortality in Older US Adults.* Am J Epidemiol, 2017. **186**(8): p. 961-969.
